# Delayed-Type Hypersensitivity to Metals of Environmental Burden in Patients with Takotsubo Syndrome – Is There a Clinical Relevance?

**DOI:** 10.1371/journal.pone.0164786

**Published:** 2016-11-08

**Authors:** Jan Manousek, Vera Stejskal, Petr Kubena, Jiri Jarkovsky, Petr Nemec, Petr Lokaj, Ludmila Dostalova, Andrea Zadakova, Marie Pavlusova, Klara Benesova, Petr Kala, Roman Miklik, Jindrich Spinar, Jiri Parenica

**Affiliations:** 1 Department of Internal Medicine and Cardiology, University Hospital Brno, Brno, Czech Republic; 2 Department of Molecular Biosciences, The Wenner-Gren Institute, Stockholm University, Stockholm, Sweden; 3 Research Centre for Toxic Compounds in the Environment (RECETOX), Faculty of Science, Masaryk University, Brno, Czech Republic; 4 Institute of Biostatistics and Analyses, Faculty of Medicine, Masaryk University, Brno, Czech Republic; 5 Centre of Cardiovascular Surgery and Transplantations, Brno, Czech Republic; 6 Faculty of Medicine, Masaryk University, Brno, Czech Republic; University of Messina, ITALY

## Abstract

**Objective:**

Takotsubo syndrome (TS) is a heart condition characterised by a sudden transient left ventricular dysfunction; its pathophysiology is probably associated with elevated levels of catecholamines but the exact mechanism is not known as yet. Literature and clinical experience suggest that TS affects persons with various comorbidities. This pilot work aims to evaluate the frequency of comorbidities with potential pathological immune reactivity, and to evaluate the potential association between TS and hypersensitivity to metals assessed by LTT-MELISA®.

**Methodology, Results:**

A total of 24 patients (23 women, 1 man) with a history of TS attack and 27 healthy controls were evaluated. Hypersensitivity was evaluated by a lymphocyte transformation test (LTT-MELISA®); a questionnaire of environmental burden was used to select evaluated metals. A total of 19 patients (79%) had at least one condition that might potentially be associated with pathological immune reactivity (autoimmune thyroid disease, drug allergy, bronchial asthma, cancer, contact dermatitis, rheumatoid arthritis). Hypersensitivity to metals was identified significantly more frequently in TS patients than in healthy controls (positive reaction to at least one metal was identified in 95.8% of TS patients and in 59.3% of controls; p = 0.003); the difference was statistically significant for mercury (45.8% and 14.8%, respectively; p = 0.029).

**Conclusion:**

Our work shows that conditions with pathological immune reactivity occur frequently in TS patients, and our data suggest a possible association between TS and hypersensitivity to metals (mercury in particular) evaluated by LTT-MELISA®. We also suggest that apart from the triggering stress factor, potential existence of other serious conditions should be considered when taking medical history of TS patients.

## Introduction

Takotsubo syndrome (TS) is a relatively rare heart condition that was first described by Sato et al. in 1990 [1]. At the time of acute attack, TS is characterised by transient left ventricular dysfunction, which most frequently affects apical segments of the left ventricle, and which cannot be explained by coronary angiography (showing a normal finding) [2]. Differential diagnosis must primarily exclude acute myocardial infarction and acute myocarditis [3]. Postmenopausal women are particularly affected, and mental stress (28%) or physical stress (36%) precedes an acute manifestation of TS [4]. Even after 25 years since the first written reference of TS, the actual cause of the condition remains unknown. Various aetiological and pathological concepts have been suggested, such as catecholamine cardiotoxicity in case of high plasma levels of these substances; this hypothesis has been supported by most clinical and experimental evidence. Changes in the microcirculation with endothelial dysfunction and coronary artery spasms, alterations in calcium-regulatory proteins, or hormonal effects have also been suggested [2,5]. It has been recently suggested to distinguish clinically between primary TS (where acute cardiac symptoms are the primary reason for seeking medical care) and secondary TS (where an acute attack develops in patients originally hospitalised for other reasons) [6]. At the present time, it is obvious that TS patients tend to have other comorbidities, often chronic ones [7], such as malignant tumours treated with chemotherapy [8], serious diseases of the gastrointestinal tract [9], psychiatric disorders [7], or neurological disorders [10–12]. Lung conditions are relatively common, such as the chronic obstructive pulmonary disease (COPD) or bronchial asthma, which are reported in 22–44% of patients [7,13]. Acute attacks of TS were repeatedly described after the administration of some drugs [14] (e.g. allopurinol [15], 5-fluorouracil [8,16], oxaliplatin [17]), or early after pacemaker implantation [18].

The everyday clinical practice suggests that not all (even serious) cases of stress burden or physical burden lead to the development of an acute attack of TS. Based on our clinical experience and published results, we were intrigued by the fact that many TS patients suffer from various chronic conditions, often associated with hypersensitivity reactions. On the other hand, hypersensitivity to metals was demonstrated in many patients with autoimmune disorders, including autoimmune thyroiditis [19], contact dermatitis [20] or connective tissue disease [21]. Hypersensitivity to metals is a type IV hypersensitivity (delayed-type hypersensitivity, DTH), which is mediated by specific T-lymphocytes. Under physiological conditions, this reaction is directed against intracellular pathogens (viruses, Chlamydia, Borrelia) and cancer cells. Most frequently, it is caused by a chronic exposure to low levels of antigen–hapten. Hypersensitivity can be evaluated by the so-called LTT-MELISA® (Memory Lymphocyte Immunostimulation Assay). It is a modified lymphocyte transformation test (LTT), which is based on the principle of antigen/allergen-specific induction of cell division in lymphocytes following contact with their respective antigens. A positive reaction in the LTT indicates the presence of antigen-specific lymphocytes (memory cells) in the patient’s blood [22,23].

We formulated the hypothesis that hypersensitivity reaction might be associated with the development of TCM. Our work aimed to describe the occurrence of conditions potentially associated with pathological immune reactivity in patients with a history of TS attack and, based on LTT-MELISA®, to evaluate the potential hypersensitivity of these patients to antigens (metal pollutants) to which they are exposed according to the questionnaire of environmental burden. These results were then compared to results of the control group of individuals with no history of TS.

## Materials and Methods

### Ethical approval of the study protocol

Written informed consent forms were obtained from all subjects before their participation in this project. The study protocol complied with the Declaration of Helsinki, and was approved by the Ethics Committee of University Hospital Brno (Brno, Czech Republic).

### Study population

The study involved patients hospitalised for an acute attack of TS from May 2006 to August 2013 at the Department of Internal Medicine and Cardiology of the University Hospital Brno. A total of 24 patients (23 women, 1 man) were admitted to the coronary acute unit with suspected acute coronary syndrome, and subsequently were diagnosed with TS according to the Mayo Clinic criteria [3]. ECG changes, hs-troponin T levels (Roche Diagnostics, Indianapolis, IN, USA, with the cut-off value at 14 ng/L), NT-proBNP (Roche Diagnostics, Indianapolis, IN, USA; with the cut-off value at 100 pg/mL), C-reactive protein (Roche, Basel, Switzerland, with the cut-off value at 5 mg/L) (all biomarkers were measured at admission), coronary angiography finding, and left ventricular function (assessed by echocardiography and left ventriculography) were evaluated in all patients. The study involved only patients with a history of TS and a documented recovery of left ventricular function. Some patients were also examined by magnetic resonance imaging (MRI) in order to rule out ischaemic aetiology or acute myocarditis. The control group was composed of 27 healthy volunteers (20 women, 7 men) who had a similar environmental burden with metals according to the questionnaire.

### Evaluation of hypersensitivity reactions by LTT-MELISA®

LTT-MELISA^®^ was performed as described previously [22]. Overall, 50 ml of venous blood were collected from each patient into tubes containing anti-coagulant and into 2 serum tubes. Blood samples were well isolated against cold and sent by overnight delivery to *Laboratoire MGD* in Switzerland for MELISA testing. Tested metals were selected according to information on metal exposure provided in patients’ questionnaires. The following metals were tested: cobalt (Co), copper (Cu), inorganic mercury (Hg), nickel (Ni), silver (Ag), tin (Sn), beryllium (Be), cadmium (Cd), chromium (Cr), gold (Au), lead (Pb), molybdenum (Mo), palladium (Pd), manganese (Mn), and titanium (Ti).

Lymphocytes were isolated from the blood by density gradient centrifugation using Ficoll-Paque^TM^ media [19] and cultivated in RPMI 1640 Medium supplemented with 10% inactivated human AB+ serum for 5 days at 37°C in a CO2 incubator. At least 2 different concentrations of various metal salts were used for each patient. Three negative controls (lymphocytes cultivated in the absence of metal salt) as well as a positive control (pokeweed mitogen, PWM) were also included. Lymphocyte proliferation in cultures was measured by the addition of isotope-labeled ^3^H thymidine for 4 hours, followed by harvesting of DNA on filters. The filters were subsequently dried, scintillation fluid was added, and radioactivity retained on filters was measured using a beta scintillation counter (Filtermat).

The stimulation index (SI) was used to evaluate the lymphocyte proliferative response. SI equals to counts per minute in metal-treated cultures divided by counts per minute in control cultures cultivated without metal salt. SI ≥ 3 was considered as positive response in our evaluation. Generally value of SI between 2 and 3 is considered as a weakly positive response, while SI ≥ 10 is considered as a strongly positive response.

The laboratory worker who evaluated LTT-MELISA® did not know which samples were taken from TCM patients or from healthy controls.

### Environmental burden

The environmental burden was evaluated by a questionnaire which assessed the exposure of a given person to metal pollutants that are mainly present in dental filling materials, prostheses and implants, the person’s smoking burden, etc. The questionnaire was evaluated by a person who did not know whether the questionnaire was completed by a TS patient or by a healthy control.

### Statistical analysis

Stimulation indices were binarised according to cut-off SI ≥ 3 (positive group), and the statistical significance of relation of these positive groups to patient categories was tested using the Fisher’s exact test. The analysis was calculated for each metal separately and for combined positive groups of all metals together. The statistical significance of differences in continuous values of stimulation indices between categories of patients was tested using the Mann-Whitney test.

## Results

The study cohort involved 24 patients with a history of TS; their basic characteristics are shown in Table 1. A total of 18 patients (75%) had apical variant of TS, while 6 patients (25%) had mid-ventricular variant of the condition. According to echocardiography, the median (5th, 95th percentile) left ventricular ejection fraction (LVEF) in the acute phase was 42% (24%, 57%); after the recovery of left ventricular function, LVEF was 65% (52%, 82%) (p < 0.001 for the difference between LVEF in the acute phase and after the recovery of left ventricular function, according to the Wilcoxon paired test). LVEF in healthy controls was 66% (56%, 73%) (p = 0.719 for the comparison of LVEF in healthy controls and TS patients after the recovery of left ventricular function, according to the Mann–Whitney U test). At admission, TS patients had high levels of NT-proBNP (median value of 525 pg/ml; 5th and 95th percentiles 150 pg/ml and 15761 pg/ml, respectively) and relatively low levels of C-reactive protein (median value of 5.98 mg/ml; 5th and 95th percentiles 0.57 mg/l and 107.33 mg/l, respectively). Mental stress, most frequently a death in the family, was the triggering factor in 6 women (25%).

**Table 1:** Basic characteristics of the TS group and the control group of healthy individuals.

	TS group (N = 24)	Controls (N = 27)
**Sex–women**	23 (96%)	20 (74%)
**Median age (years; 5**^**th**^**-95**^**th**^ **percentile)**	65.5 (41.0–75.0)	58.0 (48.0–89.0)
**Hypertension**	14 (58%)	3 (11%)
**Smoker or ex-smoker**	10 (42%)	7 (26%)
**Atrial fibrillation**	6 (25%)	0
**Corticosteroid therapy**	5 (21%)	0
**Depressive disorder**	4 (17%)	1 (4%)
**Diabetes mellitus**	4 (17%)	0
**Epilepsy**	1 (4%)	0
**Disorders potentially associated with immunopathological reaction:**		
**-Autoimunne thyroid disease**	7 (29%)	0
**-Drug allergy**	7 (29%)	3 (11%)
**-Bronchial asthma**	6 (25%)	0
**-Cancer**	4 (16%)	0
**-Contact dermatitis**	2 (8%)	7 (26%)
**-Rheumatoid arthritis**	1 (4%)	0
**At least one immunopathological disorder**	19 (79%)	8 (30%)

Table 1 shows the most frequent comorbidities. Each patient in the TS group had at least one of the mentioned comorbidities. Out of all comorbidities, we selected conditions that might potentially be associated with pathological immune reactivity (autoimmune thyroid disease, drug allergy, bronchial asthma, cancer, contact dermatitis, rheumatoid arthritis). A total of 19 patients (79%) in the TS group had at least one of these conditions.

Table 2 clearly demonstrates that environmental burden with metals is comparable between the group of TS patients and the control group.

**Table 2:** Overview of environmental burden with metals in the TS group and in the control group.

Metal of environmental burden	TS group (N = 24)	Controls (N = 27)	Fisher exact test P-value
**Ag**	24 (100%)	27 (100%)	0.999
**Al**	12 (50%)	11 (41%)	0.579
**Au**	9 (37%)	11 (41%)	0.999
**Cd**	10 (42%)	11 (41%)	0.999
**Co**	7 (29%)	7 (26%)	0.999
**Cu**	24 (100%)	27 (100%)	0.999
**Cr**	17 (71%)	17 (63%)	0.766
**Hg**	24 (100%)	27 (100%)	0.999
**Ni**	17 (71%)	17 (63%)	0.766
**Pb**	10 (42%)	11 (41%)	0.999
**Sn**	24 (100%)	27 (100%)	0.999
**Ti**	24 (100%)	27 (100%)	0.999

**Ag, Cu, Hg, Sn:** amalgam alloy; **Ni, Co, Cr:** metal-bound ceramics, metal-bound bridge; **Ni, Cr:** stainless steel; **Ti:** titan alloy of dental implants, pacemakers, implantable cardioverter defibrillators; **Al:** glass-ionomer cement; **Al, Cd, Cr, Cu, Ni, Pb:** cigarette smoke; **Al, Ti:** toothpastes, pills.

In the TS group, positive response (stimulation index SI ≥ 3) to at least one of the evaluated metals was detected in a total of 23 patients (95.8%; 22 women, 1 man). The remaining one patient (a woman) had only a weakly positive response for two metals (SI = 2.0 and 2.5). Eleven patients (46%) had a positive response to one metal, eight patients (33%) to two metals, two patients (8%) to three metals, and one patient (4%) to four metals; one patient (4%) had polyvalent allergy to six metals. Hypersensitivity reactions to nickel (11 patients, 46%) and mercury (10 patients, 42%) were most frequent, followed by hypersensitivity reactions to cobalt and silver (4 patients, 17%), cadmium and lead (3 patients, 13%), copper (2 patients, 8%), tin and chromium (1 patient, 4%).

In the control group, positive response (stimulation index SI ≥ 3) was detected in 16 persons (59.3%). Seven controls (26%) had a positive response to one metal, five controls (19%) to two metals, and four controls (15%) to three metals. Hypersensitivity reaction to nickel (9 controls, 33%) was most frequent, followed by hypersensitivity reactions to mercury and gold (4 controls, 15%), cadmium (3 controls, 11%), tin and titanium (2 controls, 7%), beryllium, chromium, cobalt and silver (1 control, 4%). Five persons from the control group did not have any response to any evaluated metal, and 6 controls had only a weakly positive response (SI = 2.0–2.9) to 1–3 metals. In general, hypersensitivity reactions were milder and less frequent in the control group when compared to the TS group.

Table 3 shows the numbers of positive LTT-MELISA® tests (SI ≥ 3) for evaluated metals in TS patients and in healthy controls. TS patients had a statistically significantly higher frequency of hypersensitivity to mercury (45.8%) than the control group (14.8%; p = 0.029).

**Table 3:** Comparison of a number of patients with positive stimulation index (SI ≥ 3) for individual metals between TS group and healthy controls.

	TS group (N = 24)		Controls (N = 27)	
	SI ≥ 3		SI ≥ 3	
Total	N (%)	Total	N (%)	P-value
**Hg** (N = 24)	11 (45.8%)	(N = 27)	4 (14.8%)	**0.029**
**Sn** (N = 17)	1 (5.9%)	(N = 19)	2 (10.5%)	0.999
**Au** (N = 10)	1 (10.0%)	(N = 9)	4 (44.4%)	0.141
**Pd** (N = 8)	0 (0.0%)	(N = 10)	0 (0.0%)	-
**Cu** (N = 18)	1 (5.6%)	(N = 25)	0 (0.0%)	0.419
**Ni** (N = 24)	11(45.8%)	(N = 27)	10 (37.0%)	0.578
**Co** (N = 18)	4 (22.2%)	(N = 27)	1 (3.7%)	0.141
**Cr** (N = 19)	2 (10.5%)	(N = 27)	1 (3.7%)	0.561
**Mo** (N = 11)	0 (0.0%)	(N = 8)	0 (0.0%)	-
**Mn** (N = 9)	0 (0.0%)	(N = 9)	0 (0.0%)	-
**Ag** (N = 23)	4 (17.4%)	(N = 27)	1 (3.7%)	0.167
**TiSO**_**4**_ (N = 18)	0 (0.0%)	(N = 13)	0 (0.0%)	-
**Cd** (N = 10)	3 (30.0%)	(N = 7)	3 (42.9%)	0.644
**Pb** (N = 10)	3 (30.0%)	(N = 7)	0 (0.0%)	0.228
**TiO**_**2**_ (N = 5)	0 (0.0%)	(N = 10)	2 (20.0%)	0.524
Combination* (N = 24)	23 (95.8%)	(N = 27)	16 (59.3%)	**0.003**

P-value of Fisher’s exact test in categorical variables.

* At least one marker has the value ≥ 3.

This statistically significant difference was evident even if we considered stimulation index ≥ 5 as the cut-off value: in this case, positive response was detected in 29.2% of TS patients, and in 3.7% of healthy controls (p = 0.019).

When evaluating the number of patients who had a positive result of LTT-MELISA® for at least one metal, we found a statistically significant higher number of individuals with such reactivity in the group of TS patients than in the control group (95.8% and 59.3%, respectively; p = 0.003).

## Discussion

Our work is the first one to point out two important issues in connection with TS: (1) a relatively frequent occurrence of conditions possibly linked to immunity, and (2) the possible link between TS and delayed-type hypersensitivity (DTH) to metals of environmental burden, evaluated by LTT-MELISA®. Apart from one patient with a weakly positive hypersensitivity reaction to two metals (stimulation index SI = 2.0 and 2.5), all other TS patients had a positive response (SI ≥ 3). This reactivity was statistically significantly more frequent in TS patients than in healthy controls. According to literature, the prevalence of hypersensitivity reactions to nickel (evaluated by patch tests) is high in the general population, estimated at 17–31% in women, and 3% in men; about 1–3% of the population are estimated to have hypersensitivity reactions to cobalt or chromium [24,25]. Evaluation of hypersensitivity reactions by LTT-MELISA® detected a positive response to one metal in 43% of healthy controls, while 18% had a positive response to two or more metals [21]. These results are comparable with those of healthy controls in our study.

According to environmental questionnaires, metal pollutants to which our patients had hypersensitive reactions corresponded to dental filling materials, but also to other environmental burden. For example, mercury, tin, silver and copper are components of dental amalgam, while alloys of nickel, chromium and cobalt are employed to shape crowns and bridges, or to bind dental ceramics. Metals such as aluminium, chromium, cadmium, copper, nickel or lead, which are present in the tobacco, might be the cause of hypersensitivity reactions in both active and passive smokers [26].

Previous studies have pointed out that there might be an association between hypersensitivity reactions to metals and autoimmune thyroiditis [19] or some other autoimmune diseases [21,27]. Depending on the individual reactivity of a specific patient (determined by a genetic predisposition), hypersensitivity reactions to metals might–or might not–develop. According to results of HLA typing, patients showing hypersensitivity reactions to metal dental filling materials had significantly increased levels of HLA-B37, B47 and DR4 antigens [28].

### Possible pathophysiological mechanism of TS in relation to hypersensitivity reactions

Development of an acute TS attack is most frequently associated with elevated levels of catecholamines [2,5], but the pathological mechanism for the development of cardiac dysfunction is not fully understood. Development of coronary microvascular spasm is one possibility. Excessively high levels of catecholamines can occur as a result of increased synthesis or reduced degradation of catecholamines, or the combination of both. During a stress response, there is a general increase in the levels of circulating catecholamines. Catechol-O-methyl transferase (COMT) is one of the enzymes involved in the degradation of catecholamines. The methionine-homocysteine cycle provides methyl groups for this process catalysed by COMT [29,30]. Moreover, methionine-homocysteine cycle is the main endogenous antioxidant system, because it is capable of binding unpaired electrons of free radicals. Cytoplasm, mitochondria and nuclei of healthy cells and tissues contain predominantly the reduced form of glutathione (GSH), accounting for up to 99% of the total glutathione pool. The reduced form of glutathione acts as an antioxidant. On the other hand, endoplasmic reticulum contains a large proportion (up to 30%) of glutathione in the oxidised form, the glutathione disulphide (GSSG). A decreased GSH-to-GSSG ratio is considered indicative of oxidative stress [29]. The methionine-homocysteine cycle also has chelation and detoxification functions [29,31]. Metal pollutants have a high affinity for thiol groups (–SH) of the reduced form of glutathione (GSH), form covalent bonds, and thus can be eliminated from the body.

**Fig 1:**
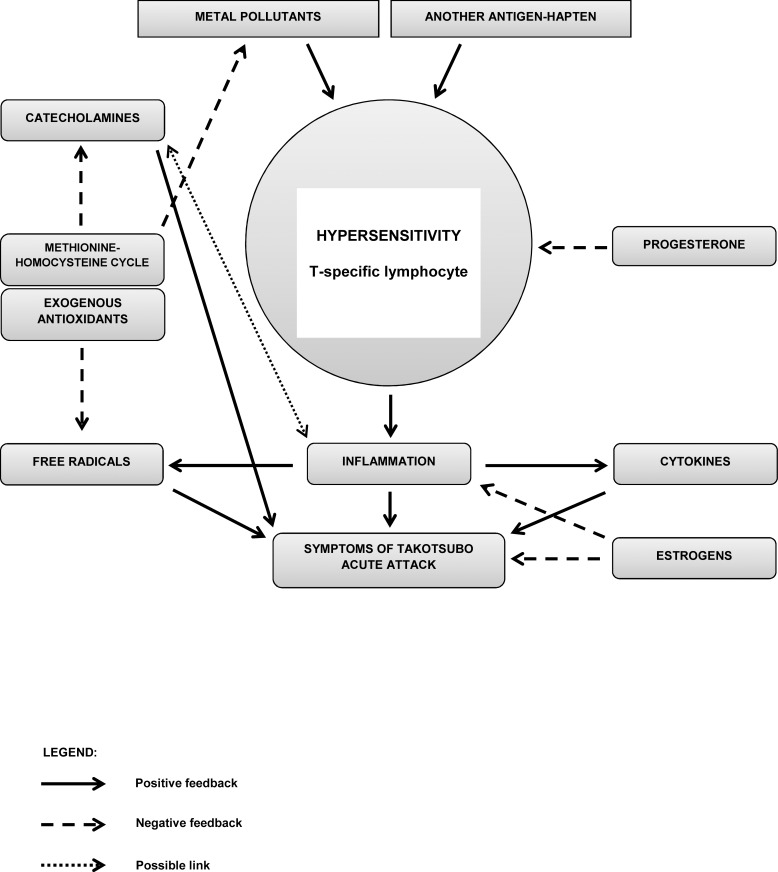
Suggested hypothesis of pathological immune mechanisms in the Takotsubo syndrome. TS is associated with increased levels of catecholamines in the acute phase of this condition. Hypersensitivity reactions to environmental burden with metals, which are mediated by antigen/hapten-specific T-lymphocytes, were proved in 96% of our patients with Takotsubo syndrome. Chronic or acute hypersensitivity reactions are accompanied by an inflammatory reaction with the production of many cytokines and free radicals (i.e., an increased oxidative stress). The methionin-homocysteine cycle is one of the main endogenous antioxidant systems. At the same time, the methionine-homocysteine cycle provides methyl groups for the degradation of catecholamines, a process catalysed by catechol-O-methyltransferase (COMT). An “exhaustion” of the methionine-homocysteine cycle as a result of chronic oxidative stress can lead to a decreased degradation of catecholamines; in a subsequent stressful situation, which is generally characterised by an increased production of catecholamines, excessively high levels of catecholamines can occur, such as those typically found in TS patients. TS develops most frequently in postmenopausal women, who have decreased levels of sex hormones, particularly oestrogens. In general, oestrogens have a cardioprotective effect (by reducing the chronotropic and ionotropic effects of catecholamines); additionally, when taking into account the potential hypersensitivity, oestrogens also have anti-inflammatory effects. On top of that, progesterone–of which levels also decrease with age–has immunosuppressive effects.

Chronic hypersensitivity reactions are accompanied by inflammatory reactions with the production of many cytokines and an increased oxidative stress [32]. A decreased performance or even the “exhaustion” of the methionine-homocysteine cycle, for example as a consequence of chronic inflammatory reaction and increased oxidative stress, as indicated above, can lead to a decreased degradation of catecholamines, among others, and therefore to an elevation of their plasma levels and an increase in their toxicity. The methionine-homocysteine cycle might represent a key link between metal pollutants, hypersensitivity reactions to metals, detoxification and antioxidant processes, and catecholamine metabolism; these processes are in some kind of dynamic equilibrium under “resting” conditions (Fig 1). No mechanisms are known to associate the hypersensitivity reaction with possible coronary microvascular spasm. Instead, vessel reactivity might possibly be influenced by the above-described chronic processes linked to an increased oxidative stress and chronic inflammation.

A disturbance of this equilibrium might apparently manifest as an acute TS attack even under less common clinical situations, for example during treatment with allopurinol, statins, or after pacemaker implantation [8–10,17,18,33]. In this context, it must be taken into account that many medications–mainly their peroral forms–contain metals as adjuvants (e.g. titanium dioxide), and that a hypersensitivity reaction can develop either to the active ingredient, or to the adjuvant.

TS mostly affects postmenopausal women (they are affected about 9 times more often than men). Possible role of female sex hormones, especially oestrogens, has therefore been suggested. They reduce chronotropic and inotropic effects of catecholamines and are cardioprotective in general [34]. When considering hypersensitive reactivity in the aethiopatogenesis of TS, progesterone is known to have immunosuppressive effects, sometimes is even referred to as natural immunosuppressive [35,36]. In menopause, decreased female hormone levels may serve as contributing factors to the development of acute TS attack. Hypersensitivity or autoimmune reactions can also lead to acute myocarditis, which might imitate TS [37]. MRI examination can be helpful in the differential diagnosis of myocarditis: late gadolinium enhancement (LGE) is typical of myocarditis, but not seen in TS [38]. Subsiding of inflammation in myocarditis can lead to an recovery of left ventricular function, similarly to that occurring in TS cases [39].

The possible link between TS development and pathological immune reactivity is documented by the case of one of our patients, who was hospitalised three times for an acute TS attack in a two-year period. A detailed examination newly revealed bronchial asthma with a severe allergic reaction to moulds. After changing residence (and thus removing exposure to the allergen) and starting asthma treatment, the TS attack did not recur over the next 4 years.

Some studies have described improvements in the condition of patients with autoimmune diseases after the removal of hypersensitivity-provoking materials, such as incompatible metal dental filling materials. This is attributed to the “downregulation” of cellular immunity [40,41].

### Limitations

Our study has several limitations. First, TS patients were examined by LTT-MELISA® 4 months to 6 years after an acute TS attack, which might seem a large time span. However, we worked on the assumption that patients’ immune reactions to metals do not change significantly, unless there is a radical change of environmental burden. Such a change was not found in the questionnaires. Nevertheless, it would be surely interesting to compare levels measured by LTT-MELISA® in patients in the acute phase with those measured in stable patients in a stable condition. It would also be interesting to evaluate other biochemical parameters in the acute phase, such as the levels of catecholamines, cortisol, corticotropin-releasing hormone, or oxidative stress markers. Second, our study was monocentric, and involved a relatively small number of evaluated persons both in the group of TS patients and in the control group of healthy individuals. Despite this limitation, it is obvious that hypersensitivity reactions to metals are very common in TS patients. Third, in both groups (TCM patients and controls), hypersensitivity reactions were only evaluated for metals selected by the questionnaire of environmental burden. Hypersensitivity reactions to metals that had not been evaluated cannot be ruled out in either of the two groups. Fourth, it is widely known that hypersensitivity reactions are attenuated by glucocorticoids. In our cohort of TS patients, five patients (20%) were on glucocorticoid therapy: four patients used glucocorticoid spray to treat COPD or bronchial asthma, one patient had systemic glucocorticoid therapy to treat rheumatoid arthritis. Although immune reactions were suppressed in these patients, hypersensitivity reactions to 1 or 2 metals (SI ≥ 3) were detected in all of them.

## Conclusion

TS is a rare condition with unclear aetiology. Our work shows that conditions with pathological immune reactivity occur frequently in TS patients and our results support the hypothesis of a possible association between TS and hypersensitivity to metal pollutants (mercury in particular) evaluated by LTT-MELISA®. The suggested pathophysiological mechanisms need to be further evaluated and verified on a large cohort of patients.

Our work also suggests that apart from the triggering stress factor, potential existence of other serious conditions should also be considered when taking medical history of TS patients.

## Supporting Information

S1 File Questionnaire of environmental burden. (DOCX)
