# Supplementary material for: Delayed-Type Hypersensitivity to Metals of Environmental Burden in Patients with Takotsubo Syndrome – Is There a Clinical Relevance?
Source: PLoS One. 2016 Nov 8;11(11):e0164786. doi: 10.1371/journal.pone.0164786 (PMC5100929; doi:10.1371/journal.pone.0164786)
Supplement: S1 File — (DOCX) [file pone.0164786.s001.docx]

# QUESTIONNAIRE OF ENVIRONMENTAL BURDEN

Name, surname:

Date of birth:

Current profession:

Past profession(s):

Diagnosed disease(s):

Contact – Telephone:

E-mail:

--------------------------------------------------------------------------------------------------------------------------------------

## 1. Dental record.

1.1. Current dental fillings or implants – material(s), number, exposure time.

Amalgam:

Gold crown/bridge:

Metal-bound ceramics:

Titanium crown/bridge:

Cobalt-chrome-nickel crown/bridge:

Ceramics:

Composites:

1.2. Root fillings – material(s), number, exposure time.

Amalgam:

Gutta-percha:

Calcium hydroxide:

Other:

1.3. Do you have any dental implants? (titanium, zirconium, other):

1.4. Have you undergone any dental fillings replacement or teeth reconstruction procedure? –

material(s), number, dates.

1.5. Which material has been replaced? (Gold, cobalt-chrome-nickel crown/bridge, metal-bound

ceramics, titanium crown/bridge, composites, ceramics).

1.6. Is the replacement temporary or permanent?

1.7. Have you ever had dentures or braces?

Temporarily – when?

Permanently – since when?

1.8. Have you experienced any side effects when wearing them? (Metallic taste, pain or swelling in

your mouth, bleeding gums, burning, itching or irritation in your mouth).

1.9. Do your gums bleed when you brush your teeth?

- rarely

- frequently

- regularly.

1.10. Have you experienced teeth grinding while sleeping? Since when?

## 2. Metal exposure.

## 2.1. Have you ever had any metal implants in your body ( screws, pins, staples, pacemaker,

cardioverter-defibrillator, stent, occluder etc?).

2.2. Do you have any tattoos? Since when, how extensive?

2.3. Have you been exposed to metals in your work place (foundry, plating, machining,

sandblasting of metals etc.)? Which metals and how many months/years??

2.4.1. Are you a current smoker (cigarettes, cigars, pipe)? How long have you been smoking? How

many cigs per day/week/month?

2.4.2. Are you an ex-smoker? How many years did you smoke for? How many cigs per

day/week/month?

2.4.3. Are you being exposed to passive smoking at your work place or at home?

2.5. **Vaccines.**

2.5.1. Have you ever been given a Gamma globulin vaccine? Yes – No – I don´t know.

2.5.2. Have you ever received a flu vaccine? Yes – No – I don´t know.

2.5.3. Have you ever experienced an adverse reaction to any vaccine in the past? Please specify

when, what type of vaccine, symptoms.

2.6. **Contact lenses.**

- Have you ever used soft contact lenses?

- Have you experienced any side effects?

2.7. **Cosmetics.**

- Do you use cosmetics (deodorants, antiperspirants, aftershave, makeup, sunscreens)? Which

and how often?

2.8. **Earrings and piercing.**

- Do you wear earrings or are you pierced? What material was used?

- Have you experienced discomfort/intolerance of earrings or piercing? Please specify which

and since when.

2.9. Have you ever suffered from skin irritation when in contact with jeans buttons, wristwatches,

rings, jewellery or other such items? Which and since when?

2.10. Have you ever received any injections for allergy treatment (allergy shots)?

2.11. **Diet.**

- Do you eat fish and seafood? Which and how often?

2.12. Do you chew gum? How often?

2.13. What brand of toothpaste do you use?

2.14. Do you eat crunchy-coated or multi-colored confectionery? Which and how often?

2.15. Have you ever undergone a skin test/patch test for metal allergy? Why? When and what was

the result?

2.16. Have you ever lived close to a metallurgical factory, industrial plants,

motorway/highway, airport, crematorium, dental office?

**3. For women.**

3.1. Have you ever had breast implants? Which and how long?

3.2. Have you ever used an IU device? Which and how long?

**4. Allergies and illnesses.**

4.1. Are you allergic to any antibiotics (penicillin, sulfonamides etc)? Which?

4.2. Do you have any food allergies? Which foods, since when? Please specify symptoms.

4.3. Do you have any other allergies?

4.4. Do you have any diagnosed illness/illnesses?

4.5. **Your family health.**

Does anyone in your family suffer from the following disease? How long?

- Allergies.

- Autoimmune disease.

- Skin disease.

- Heart and vascular disease.

- Diabetes.

- Cancer/tumor.

- Chemical sensitivity.

- Psychological illness.

- Neurological disease.

- Other.

4.6. Do you currently take any medication? Which? How long?

4.7. Do you currently take vitamins or mineral supplements? Which? How long?

4.8. Have you experienced any side effects from these supplements?

4.9. Have you been bitten by a tick within the past 5 years? When?

- Have you been tested for any infectious tick-borne disease, such as Lyme/Borrelia? Which

tests and what were the results?

**Thank you very much for your cooperation.**
